# Caries Prevalence Evolution and Risk Factors among Schoolchildren and Adolescents from Valencia (Spain): Trends 1998–2018

**DOI:** 10.3390/ijerph17186561

**Published:** 2020-09-09

**Authors:** Teresa Almerich-Torres, José María Montiel-Company, Carlos Bellot-Arcís, José Enrique Iranzo-Cortés, José Carmelo Ortolá-Siscar, José Manuel Almerich-Silla

**Affiliations:** Stomatology Department, University of Valencia, 46010 Valencia, Spain; jose.maria.montiel@uv.es (J.M.M.-C.); carlos.bellot@uv.es (C.B.-A.); j.enrique.iranzo@uv.es (J.E.I.-C.); jose.c.ortola@uv.es (J.C.O.-S.); jose.m.almerich@uv.es (J.M.A.-S.)

**Keywords:** caries prevalence, caries epidemiology, risk factors, surveillance, schoolchildren, cross-sectional survey, ICDAS II, socioeconomic status

## Abstract

The aim of this study was to determine the caries status and risk factors in the schoolchildren of Spain’s Valencia region in 2018 and to compare them to the 20-year evolution of caries indicators in the region. A cross-sectional survey was conducted with 1722 children and adolescents aged between 6 and 15 using cluster sampling. Caries status, using International Caries Detection and Assessment System II (ICDAS II) criteria, and sociodemographic variables were recorded. To ensure the comparison with previous studies using WHO caries criteria, the cut-off point was established at ICDAS II code 4. Caries prevalence was found to be 37.4% and the decayed and filled teeth index (dft) was 1.23 at 6 years for deciduous dentition (DD). In permanent dentition (PD) at 12 years, caries prevalence was 30.1% with a 0.66 decayed, missing and filled teeth index (DMFT), and at 15 years, prevalence was 44.6% and DMFT was 1.21. Socioeconomic status poses a major risk factor for caries prevalence in deciduous dentition; it is 1.8 times higher in the lowest socioeconomic group. Deciduous dentition status has worsened in the most recent eight-year period, whereas in permanent dentition the 12- and 15-year values are similar to those of the 2010 survey. Evolution analysis suggests that community dental care programs be enhanced, involving preventive activities staring at the first year and targeting disadvantaged groups.

## 1. Introduction

Dental caries is a disease that affects the hard tissues of the tooth, caused by the interaction over time of microorganisms present in dental plaque and fermentable carbohydrates in the diet, mainly in the form of sugars such as sucrose [1]. Current etiological knowledge considers it a non-communicable disease (NCD) [2] and related with determinants of health such as genetics and biology, social and physical environment, health behaviors, and dental and medical care [3]. Dental caries continues to be one of the most prevalent chronic diseases in both children and adults. The evolution of carious lesions results in cavities in the teeth that cause pain and, ultimately, loss of teeth, which results in a major impact on basic physiological functions such as eating, sleeping, speaking, and personal productivity. This can become an important barrier to achieving a general state of good health [1,4].

Oral health surveys are tools that facilitate the gauging of the oral health status of populations; such surveys can be used by researchers to monitor changes in morbidity levels, monitor trends, and determine the treatment needs of a given population [5]. Furthermore, they allow us to assess the extent to which existing dental services respond to the needs of the population, the number of prevention and restorative programs required, and the resources necessary to implement or maintain oral health programs [6,7]. The World Health Organization (WHO) recommends that these surveys be carried out periodically to monitor the evolution of oral health and analyze the changes that have occurred over time in populations [5]. In the Spanish region known as Comunidad Valenciana (Valencian Community, CV), epidemiological surveys were carried out in children and young people in 1986, 1991, 1998 [8], 2004 [9], and 2010 [10]. Over time, the methodology of these surveys was adapted to be as standardized as possible. We cannot ignore that the traditional concept of caries as a multifactorial communicable disease has evolved [11] and, therefore, the diagnostic and measurement criteria of the disease has had to adapt accordingly. The survey carried out in 2010 in the Comunidad Valenciana [10] was the first in Spain to use the International Caries Detection and Assessment System II (ICDAS II) criteria in this type of survey. In 2018 we maintained these diagnostic criteria for the collection of caries status and, in addition, we incorporated the recommendations of a recent 2018 publication: The Brussels Statement on the Future Needs for Caries Epidemiology and Surveillance in Europe [12], which describes the methodology that future epidemiological studies should comply with; this includes the use of standardized caries diagnostic criteria (ICDAS), the use of appropriate reporting measures that reflect changes in the distribution of caries across populations, the consideration of the inequalities in health in the design of the studies, and the collection of variables related to the socioeconomic level, habits, and the quality of life. The present study shows the oral health evolution of the child and youth populations of the Comunidad Valenciana (Spain) over the last 20 years and analyzes the relationships with the socioeconomic and health system changes that may have influenced this evolution.

## 2. Materials and Methods

A cross-sectional epidemiological study was carried out following the recommendations published in the report The Brussels Statement on the Future Needs for Caries Epidemiology and Surveillance in Europe [12] for conducting oral health surveys, in the cohorts of schoolchildren from the Comunidad Valenciana of 6, 12, and 15 years of age.

### 2.1. Sample

A controlled bias sample was taken, relying on the experience obtained in the previous studies in 2004 [9] and 2010 [10] in this region. Because the individuals in the population are organized into small groups (classrooms), and they are schoolchildren, a cluster sampling approach was chosen.

The sample size calculation was based on the caries prevalence results from our previous study in 2010 [10]. It was estimated that a random sample of 505 individuals is a representative quantity for the 6-year cohort, 566 individuals for the 12-year cohort, and 592 individuals for the 15-year cohort with a 95% confidence level and based on a ± 4% degree of precision. Of the 2144 clusters made up of the students’ classrooms, 59 were selected: 19 were randomly selected for 6-year-old, 20 for 12-year-old, and 20 for 14 to 15-year-old.

### 2.2. Calibration

The fieldwork was carried out by three examination teams, each comprising one examiner (dentist) and one annotator (assistant). To ensure reliability and validity of the results, the examiners performed a prior calibration. Before commencing the examinations, an informative meeting on the characteristics of the study was held, in which an examination manual was presented to the 3 examiners and 3 annotators, containing the diagnostic criteria and the data to be collected, in addition to the examination sheets that would be used in the field work. Once the content was studied by the examiners and annotators, three theoretical sessions were held for the sharing and resolution of doubts or conflicts. Once the doubts were clarified, a common calibration exercise was organized. This exploration was performed with 15 children between the ages of 6 and 12, making up a total of 289 teeth, which were examined by each examiner. Diagnostic concordance was studied using the combined ICDAS II criteria that considers 4 stages of caries (healthy, non-cavitated caries, moderate caries, and extensive caries) with respect to an experienced examiner who acted as a gold standard. The 3 examiners showed a good to exceptionally good level of agreement, according to the Landis and Koch scale, with a linear weighted Kappa index of 0.87, 0.76, and 0.82, respectively.

### 2.3. Material

The material used for the examination consisted of a WHO-type periodontal probe (CP-11.5B Screening Probe, Hu-Friedy^®^-Chicago, IL, USA), and a flat No. 5 intraoral mirror. In each examination, a pair of nitrile gloves was used to avoid any possible allergic reactions among participants, in addition to disposable surgical masks.

### 2.4. Examinations

The examinations were carried out at schools and high schools (public and private), generally in a multitasking classroom with natural light. The examiners were trained to perform the data collection under the best possible lighting, location, and ergonomic conditions. To improve the examination conditions, each explorer had a white light emitting diode (LED) headlamp. In the absence of a dental chair, the subjects were examined while they were sitting in a chair with their backs straight and their heads tilted backwards. The examiner either stood or sat facing the child sitting in another chair. While the examiner carried out the examination, the annotator filled out the data collection form. All scans were carried out between April and October 2018.

To carry out the examinations of the schoolchildren, detailed information about them was previously given to the parents or legal guardians, in a document that was required to be signed with their consent to participate in the study. Once the signed authorizations were received from the parents or legal guardians, the dental examination was performed, after which individualized reports with the findings were sent to the parents or guardians. This study was approved by the Ethical Committee for Human Research of the University of Valencia with the reference number H1510648717945, thereby respecting the ethical principles of the Declaration of Helsinki and complying with current regulations regarding data protection.

### 2.5. Caries Diagnosis Criteria

The caries status was assessed according to the ICDAS II criteria [13] with two digits; the first identifies the presence of any type of restoration or seal and the second registers the caries code for each tooth. All dental surfaces of each of the present and measurable teeth were explored: five surfaces for the premolar and molar teeth and four for the incisors and canines. In the present study, due to the difficulties of obtaining an adequate dryness level of the dental surfaces during the examination, we decided to make use of the epidemiological variant of ICDAS II criteria, which allows the fusion of codes 1 and 2 [14].

To allow comparability of the results obtained using the ICDAS II criteria with studies using the WHO criteria, the cut-off point was established in code 4 of ICDAS-II [15]. Following from this, ICDAS-II values 4, 5, and 6 comply with the WHO criteria cavity definition.

### 2.6. Study Variables

The variables calculated for the study were caries prevalence (ICDAS II 2-6/C-G > 0 and ICDAS II 4-6/E-G > 0), decayed and filled teeth index (dft) ICDAS II C-G, df.t ICDAS II E-G, decayed and filled surface index (dfs) ICDAS II C-G, dfs ICDAS II E-G, decayed, missing and filled teeth index DMFT-ICDAS II 2-6, DMFT ICDAS II 4-6, decayed, missing, and filled surface index (DMFS) ICDAS II 2-6, and DMFS ICDAS II 4-6. The restoration, morbidity, mortality, and Significant Caries Index (SCI) [16] indices were calculated from the codes E-G/4-6, for both deciduous and permanent dentition. 

### 2.7. Socioeconomic Status

The assessment of socioeconomic status was based on the parents’ occupation [17], in which the socioeconomic status of the child was considered to be the higher of those of the two parents. The categories were grouped into low, middle, and high social class. Classes V and IVb were considered low socioeconomic status, IVa and III middle socioeconomic status, and I and II high socioeconomic status.

### 2.8. Statistical Analysis

The data collected in each examination form were stored by each examination team in a Microsoft^®^ Excel^®^ program (Washington, DC, USA) database. Statistical analysis was performed with the IBM^®^ SPSS v 24 program (New York, NY, USA). A univariate descriptive statistical analysis was performed obtaining the means for the quantitative variables and proportions for the dichotomous or categorical variables, in addition to their 95% confidence intervals. In relation to bivariate statistics, the Student’s t-test and ANOVA were used for the comparison of means, after the normality level of the distributions was confirmed with the Kolmogorov–Smirnoff test; the chi-square test was used in the comparison of proportions. The level of significance was established for *p* < 0.05.

## 3. Results

The final sample of the study was 1722 children and adolescents from 59 schools throughout the Comunidad Valenciana; 556 children were 6 years old, 632 were 12 years old, and 534 were 15 years old, with a similar distribution of sexes: 48.08% were boys and 51.92% were girls. The prevalence and caries indices were calculated using the ICDAS II 4-6/E-G codes, equivalent to the WHO criteria, and the ICDAS II 2-6/C-G codes, to enable the comparison of the results with surveys carried out under ICDAS II criteria. The results for the prevalence of caries are shown in Table 1.

The dft index (ICDAS II E-G > 0) was 1.23 at 6 years, and the DMFT index (ICDAS II 4-6 > 0) was 0.66 at 12 years and 1.21 at 15 years. When accounting for all caries codes (ICDAS II 2-6/C-G), the dft index was 1.68 at 6 years, and the DMFT was 1.99 at 12 years and 2.85 at 15 years. All indicators maintain a 95% confidence interval (CI). Table 2 and Table 3 present a summary of the ICDAS-II codes and the most important caries indicators obtained using both ICDAS II 4-6/E-G and ICDAS II 2-6/C-G diagnostic criteria, in overall consideration of tooth (Table 2) and surfaces, respectively (Table 3).

To ensure comparability with other studies, the ICDAS II 4-6/E-G criteria were maintained to calculate dental restoration, morbidity, and mortality rates. The restoration rate was 26.8% at 6 years for deciduous dentition (DD), 71.2% at 12 years, and 85.1% at 15 years. The morbidity rate was 73.2% at 6 years (for DD), 27.3% at 12 years for permanent dentition (PD) and 14.9% at 15 years. Dental mortality barely reached 1.5% at 12 years. The Significant Caries Index (SCI) [16], calculated for ICDAS II 4-6/E-G, was 1.97 at 12 years (*n* = 211) for PD and 3.56 at 6 years for DD (*n* = 185). The 80/20 phenomenon was also observed in our results: in deciduous dentition, 79.1% of caries were accumulated by only 19.8% of children aged 6 years. In permanent dentition (PD), 81.1% of caries at 12 years were concentrated in the group accounting for 17.7% of schoolchildren. 

The analysis of the prevalence of caries and of the different indices, both when considering all the ICDAS-II codes 2-6, and when only the ICDAS-II codes 4-6 were considered, in the three age groups, showed no statistically significant differences with respect to sex in any of the comparisons. Nor were statistically significant differences found in the immigrant population with respect to the local population.

Regarding the distribution by socioeconomic status, the percentage of children from the highest socioeconomic status was 34.7%, while 39.0% was from the middle-level socioeconomic status, being the predominant social class, and 26.2% from the lowest socioeconomic status. Regarding the relationship of caries with socioeconomic status, there are statistically significant differences at the age of 6 years, both for ICDAS 2-6/C-G and ICDAS 4-6/E-G. Children of 6 years from the lowest socioeconomic status had a dft of 1.69, whereas those of the high status had a dft of 0.68. Those from the low-status group had 2.48 times more cavities and a prevalence of caries 1.8 times higher than those belonging to the high socioeconomic group (*p* = 0.00). No statistically significant differences were observed in the 12- and 15-year cohorts (Table 4).

To compare the evolution of the caries indicators in the period between 1998 and 2018, we collated the information from the studies as presented in Table 5.

## 4. Discussion

Conducting epidemiological studies in schoolchildren continues to be a good instrument to monitor the evolution of caries indicators and the association with socioeconomic variables; however, carrying them out has become increasingly complex due to the difficulties of their implementation in the school setting and the issue of safeguarding the participation rights that may be exercised by the participants and/or their legal guardians. In this study we obtained positive informed consent and this may have been the reason why we found a rejection rate of 18.5%, although the number of schoolchildren examined in each of the age cohorts exceeded the sampling forecasts. The rejection rate recorded in the 2010 survey [10] was 21%.

The use of the ICDAS II caries diagnostic criteria [13] in epidemiological surveys usually encounters the difficulty of not having an effective tooth drying scenario and this was solved using an approved epidemiological modification that allows merging codes 1 and 2 [14]. From the exploration with ICDAS-II criteria, the caries index was reconstructed considering all of the stages (ICDAS 2-6/C-G), or only the most advanced lesions (ICDAS 4-6/E-G) that we matched to the caries WHO criteria. The establishment of this cut-off point has generated controversy in the literature [18,19], but it has been a priority objective of this research to be able to infer a reliable comparison of the results obtained in 2018 with those obtained in the 2010 study [10].

Diagnosing carious lesions from their earliest stages is consistent with the recent concept of caries as a non-communicable disease (NCD) in which complete removal of carious tissue is not necessary [20]. The availability of more sensitive indicators in the detection of early lesions commences with the onset of the paradigm shift in the treatment of caries disease; it entailed moving from a purely operational approach to a more preventive one, based on non-operative treatment and therapies and minimal intervention when caries removal is indicated [21,22], thus fulfilling the objectives of preserving the maximum of healthy dental structure, preventing enamel demineralization, and promoting natural tooth repair processes [23]. The recommendations published in the report The Brussels Statement on the Future Needs for Caries Epidemiology and Surveillance in Europe [12] for conducting epidemiological oral health surveys are clear in this regard and indicate the need to use the ICDAS diagnostic criteria, and its evolution in the ICCMS (International Caries Classification and Management System), designed to be used in four approaches (clinical, education, research, and public health) [24], with regard to the classic WHO criteria. The use of diagnostic criteria that consider the lesion from the initial stage shows a high percentage of non-cavitated caries (stage or ICDAS code 3 in most cases) that are not being considered and that have a significant risk of becoming cavitated lesions in a short period of time if they do not receive adequate care and attention [25], and therefore an inability to act with this preventive or minimal intervention approach. As observed in Table 2, at the age of 6, the dft index increases by 36% when all of the codes are considered (C to G), while the DMFT index, when all the codes are considered (2 to 6), triples at the age of 12 and doubles at the age of 15. Therefore, a high number of carious lesions that are not considered when using the WHO criteria is evident.

Socioeconomic status appears as a proven caries risk factor with a strong association, especially in developed countries [26]. Our results also reflect this factor; at 6 years, children classified as belonging to a high socioeconomic status show a dft index of 0.68, in the middle socioeconomic status the value is 1.24, whereas in those with low socioeconomic status, it increases to 1.69; in other words, children of 6 years with a low socioeconomic status have 2.48 times more cavities than children with a high socioeconomic status. These differences related to socioeconomic status are not reflected in the permanent dentition of the cohorts of 12 and 15 years, but it is again reflected in the prevalence of caries in the deciduous dentition stage at 6 years. A share of 25.8% of high socioeconomic status children present cavities, while that number rises to 38.5% in children with mid-level socioeconomic status; the prevalence reaches 46.6% in populations with lower socioeconomic status. This is particularly worrying data and requires the urgent adoption of inclusive policies in this regard. In our previous 2010 study [10], the repercussions of social differences in caries indices were also found, in addition to the 6-year cohort, in the 15-year cohort.

This study allows us to analyze the evolution of caries indicators over the last 20 years in this region by comparing studies that were carried out by our research group, although the most recent two studies used an examination methodology based on the ICDAS II criteria; in this regard, we made an adjustment that allowed us to compare it with previous data in which we had used the WHO criteria. As shown in Table 5, in 2018 the prevalence of caries in deciduous teeth at 6 years in Comunidad Valenciana was 37.4%, marking a pronounced increase compared to figures from 2010, 2004, and 1998. At 12 years, on the contrary, the prevalence of caries in permanent teeth shows a clear downward trend to 30.1%. Finally, at 15 years of age, the current prevalence of caries in permanent teeth stood at 44.6%, remaining stable and at levels similar to 2010, consolidating the decrease in the trend when compared with the high values recorded in 2004 (55.9%) and 1998 (69.3%).

**Figure 1:**
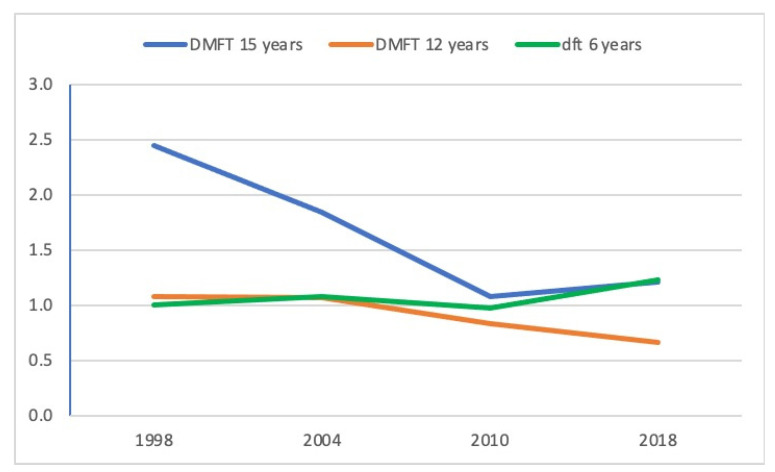
Evolution of caries indices between 1998 and 2018.

If we observe the evolution in caries indices (Figure 1), both for deciduous dentition (DD) at 6 years and for permanent dentition (PD) at 12 and 15 years, we also find an overall downward trend in the three age cohorts since 1998. However, in the most recent period, this decreasing trend was interrupted in the 6- and 15-year cohorts. In the DD at 6 years, the dft was 1.23, compared to 0.98 in 2010, 1.08 in 2004, and 1.00 in 1998. At 15 years, the DMFT index in 2018 was 1.21, a value slightly higher than that found in 2010 (1.08), but lower than in 2004 (1.84) and in 1998 (2.45). In the 12-year cohort, the downward trend of the DMFT index continues (0.66 in 2018, compared to 0.83 in 2010, 1.07 in 2004, and 1.08 in 1998), even observing lower values than those obtained for the Spanish population [27].

The Significant Caries Index (SCI) is an indicator that is used as a complement to the DMFT index to assess the skewed distribution of caries [16]. In 2018, the SCI at 12 years old was 1.97 and a comparison with previous data obtained in our community (2.36 in 2010 and 2.94 in 2004) indicates a downward trend in this index and in the so-called “80/20 phenomenon” of caries concentration in this age cohort, although this phenomenon is still maintained as shown in our results.

In our opinion this general improvement in the oral health status of schoolchildren can be related to an improvement in general living conditions in the last 20 years, with an increase in the Gross Domestic Product (GDP) of 177% in this period. However, other factors may have had a greater influence on this evolution, such as the introduction in 1986 of a Children’s Oral Health Programme, which was redefined and expanded in 1996, introducing health education and health promotion activities in schools. These reforms integrated oral health content with general health content in a cross-sectional way, performing fluoridated mouth rinses at school and performing dental check-ups at public health system clinics, which include the application of professional fluoride and the placement of pit and fissure sealants, free of charge for the population aged 6 to 15 years. Starting in 2008, community dental clinics included new restorative dentistry benefits (restoration and endodontic procedures), although exclusively related to permanent dentition. Currently, the 109 community dental clinics provide preventive and restorative services in permanent dentition to the 477,000 children between the ages of 6 and 15, absolutely free, although there are important differences in the distribution ratio of children in the different clinics. This may explain why we found a low rate of use of public consultations in our study. Only 25.2% of 12-year-old children participating in the study had attended a public system consultation and at 15 the figure was still lower, at 17.8%.

Another important factor to consider when analyzing the evolution of caries indicators in this 20-year period is the progression experienced by the number of dentists in the Comunidad Valenciana. Since 1998, when the dentist to inhabitant ratio was 1/2759, the ratio improved to 1/1219 in 2018 (data from the General Dental Council of Spain), representing a 279% increase in the number of dentists. Given the low use of public consultations, it may be that the use of private dental clinics may be related to the notable increase in the restoration rates observed, from 45.3% in 1998 to 71.2% in 2018 at age 12 (Table 5); nonetheless, the provision of these services, with payment for the medical service provided, may not be the most advisable option for children.

The evolution of the caries indicators in deciduous dentition, contrary to what is observed in permanent dentition, has not manifested a clear improvement in the 20-year period, in spite of a slight rebound in the most recent period between 2010 and 2018. It appears that the start of community dental care at 6 years has a perceptible impact; a recommendation would be that children’s dental care programs should be started earlier and with special emphasis given to preventive measures.

## 5. Conclusions

The evolution of caries indices in children and adolescents in the Spanish region known as Comunidad Valenciana over the last 20 years has shown a favorable evolution at 12 and 15 years in permanent dentition. However, the trend in deciduous dentition has remained stable, with a slight deterioration in the past eight years. The existence of a community oral health program, which includes restoration services from the age of 6, together with the large increase in private dentists, may be the most important cause of the significant increase in restoration rates found at 12 and 15 years. Socioeconomic status continues to be a major caries risk factor, especially for deciduous dentition in 6-year-old children. The evolution analysis suggests the need to strengthen community dental care programs, with the implementation of preventive activities, from the first year of life and with special attention to the most disadvantaged groups.

## Figures and Tables

**Table 1:** Caries prevalence in the three age groups.

Caries Prevalence	6 Years *	12 Years	15 Years
dft/DMFT ICDAS-II E-G/4-6 > 0	37.4%	30.1%	44.6%
	(33.5–41.5%) **	(26.6–33.7%)	(40.4–48.8%)
dft/DMFT ICDAS-II C-G/2-6 > 0	49.1%	58.2%	65.9%
	(45.0–53.2%)	(54.3–62.0%)	(61.8–69.8%)

* Deciduous dentition (DD); ** Confidence interval (CI). International Caries Detection and Assessment System (ICDAS); Decayed, missing and filled teeth index (DMFT).

**Table 2:** Indicators by tooth and age group.

ICDAS II Caries Codes	6 Years *	12 Years	15 Years
ICDAS 2-C	0.26	0.90	1.08
	(0.20–0.32)	(0.78–1.02)	(0.93–1.23)
ICDAS 3-D	0.19	0.43	0.56
	(0.15–0.24)	(0.35–0.50)	(0.46–0.66)
ICDAS 4-E	0.42	0.13	0.14
	(0.34–0.50)	(0.09–0.16)	(0.09–0.19)
ICDAS 5-F	0.31	0.02	0.02
	(0.22–0.39)	(0.01–0.03)	(0–0.03)
ICDAS 6-G	0.17	0.03	0.01
	(0.10–0.23)	(0.01–0.04)	(0–0.03)
Missing teeth		0.01	0.00
		(0–0.02)	(0–0.01)
Filled teeth	0.34	0.47	1.03
	(0.26–0.41)	(0.39–0.56)	(0.89–1.18)
dft/DMFT ICDAS-II C-G/2-6	1.68	1.99	2.85
	(1.48–1.88)	(1.79–2.18)	(2.59–3.11)
dft/DMFT ICDAS-II E-G/4-6	1.23	0.66	1.21
	(1.05–1.40)	(0.56–0.75)	(1.05–1.36)

* Deciduous dentition (DD); C, D, E, F, G are the ICDAS II scale for deciduous dentition.

**Table 3:** Indicators by surface and age group.

ICDAS II Caries Codes	6 Years *	12 Years	15 Years
ICDAS 2-C	0.30	1.02	1.24
	(0.23–0.37)	(0.87–1.14)	(1.05–1.44)
ICDAS 3-D	0.27	0.50	0.63
	(0.20–0.34)	(0.41–0.59)	(0.51–0.75)
ICDAS 4-E	0.58	0.15	0.16
	(0.46–0.70)	(0.10–0.19)	(0.10–0.23)
ICDAS 5-F	0.50	0.04	0.02
	(0.36–0.64)	(0.02–0.07)	(0–0.04)
ICDAS 6-G	0.49	0.06	0.05
	(0.28–0.69)	(0.02–0.10)	(0–0.13)
Missing surfaces	-	0.06	0.01
		(0–0.12)	(0–0.03)
Filled surfaces	0.69	0.58	1.28
	(0.52–0.87)	(0.48–0.69)	(1.07–1.48)
dfs/DMFS ICDAS-II C-G/2-6	2.83	2.39	3.40
	(2.43–3.23)	(2.13–2.66)	(3.04–3.76)
dfs/DMFS ICDAS-II E-G/4-6	2.26	0.88	1.53
	(1.89–2.63)	(0.72–1.04)	(1.29–1.76)

* Deciduous dentition (DD).

**Table 4:** Main indicators of caries according to ICDAS II 4-6/E-G criteria, by age cohort and socioeconomic status.

ICDAS II4-6/E-G	6 Years			12 Years			15 Years		
	*n* = 556			*n* = 632			*n* = 534		
	High	Middle	Low	High	Middle	Low	High	Middle	Low
	*n* = 159	*n* = 221	*n* = 176	*n* = 243	*n* = 236	*n* = 153	*n* = 198	*n* = 214	*n* = 122
DMFT	0.07	0.12	0.11	0.64	0.72	0.59	1.13	1.29	1.19
	0.02–0.14	0.07–0.18	0.04–0.18	0.48–0.79	0.53–0.89	0.42–0.77	0.9–1.36	1.03–1.55	0.84–1.54
	*p* = 0.54			*p* = 0.65			*p* = 0.67		
DMFS	0.11	0.33	0.35	0.91	0.99	0.69	1.43	1.53	1.67
	0.01–0.20	0.15–0.51	0.10–0.59	0.64–1.17	0.69–1.29	0.48–0.90	1.1–1.74	1.22–1.85	0.95–2.4
	*p* = 0.16			*p* = 0.35			*p* = 0.76		
dft	0.68	1.24	1.69	0.06	0.08	0.12	0.01	0.01	0.02
	0.46–0.91	0.96–1.53	1.32–2.05	0.02–0.09	0.01–0.15	0–0.25	0–0.01	0–0.01	0–0.04
	*p* = 0.00 *			*p* = 0.56			*p* = 0.43		
dfs	1.21	2.20	3.29	0.10	0.16	0.16	0.01	0.01	0.03
	0.76–1.64	1.63–2.77	2.48–4.10	0.03–0.17	0.04–0.29	0.01–0.32	0–0.02	0–0.02	0–0.08
	*p* = 0.01 *			*p* = 0.97			*p* = 0.56		
Caries Prevalence (PD) **	5%	10%	6.8%	30%	29.7%	30.7%	45.5%	45.8%	41%
	2.6–9.6	6.7–14.6	3.9–11.5%	24.6–36.1	24.2–35.8	23.9–38.4	38.7–52.4	39.3–52.5	32.7–49.9
	*p* = 0.18			*p* = 0.98			*p* = 0.66		
Caries Prevalence (DD) **	25.8%	38.5%	46.6%	4.5%	4.2%	3.9%	0.5%	0.5%	1.6%
	19.6–33.1	32.3–45	39.3–53.9	2.5–7.9	2.3–7.6	1.8–8.3	0.1–2.8	0.1–2.6	0.5–5.8
	*p* = 0.00 *			*p* = 0.96			*p* = 0.43		
Caries Prevalence Overall	30.8%	41.6%	47.7%	33.3%	32.2%	32%	46%	46.3%	42.6%
	30.8–24.2	35.3–48.2	40.4–55.1	27.7–39.5	26.6–38.4	25.2–39.8	39.2–52.9	39.7–52.9	34.2–51.5
	*p* = 0.01 *			*p* = 0.95			*p* = 0.79		

* *p* < 0.05. ** Permanent Dentition (PD) and Deciduous dentition (DD).

**Table 5:** Evolution of the main indicators of dental caries between 1998 and 2018.

Age	Indicator	1998	2004	2010	2018
6 years	dft	1.00	1.08	0.98	1.23
		-	(0.89–1.27)	(0.79–1.17)	(1.05–1.40)
	dfs	1.98	2.14	1.44	2.26
		-	(1.71–2.57)	(1.07–1.79)	(1.89–2.63)
	Caries Prevalence DD *	32.8%	32.2%	30%	37.4%
		-	(28.1–36.4%)	(26–34.2%)	(33.5–41.5%)
	Restauration Index	12.00%	24.50%	14.30%	26.80%
12 years	DMFT	1.08	1.07	0.83	0.66
			(0.92–1.22)	(0.69–0.96)	(0.56–0.75)
	DMFS	1.65	1.56	1.27	0.88
		-	(1.31–1.81)	(1.04–1.49)	(0.72–1.04)
	Caries Prevalence PD *	45.9%	42.5%	37.7%	30.1%
		-	(37.9–47.0%)	(33.4–42.3%)	(26.6–33.7%)
	Restauration Index	45.30%	32.70%	59.00%	71.20%
15 years	DMFT	2.45	1.84	1.08	1.21
		-	(1.60–2.08)	(0.91–1.24)	(1.05–1.36)
	DMFS	3.69	2.57	1.64	1.53
		-	(2.17–2.97)	(1.36–1.92)	(1.29–1.76)
	Caries Prevalence PD *	69.3%	55.9%	43.6%	44.6%
		-	(50.8–60.7%)	(39.1–48.4%)	(40.4–48.8%)
	Restauration Index	56.30%	45.00%	71.30%	85.10%

* Deciduous dentition (DD) and Permanent Dentition (PD).
